# Skeletal muscle metastasis from gastric carcinoma: a systematic review of published case reports and case series over six decades

**DOI:** 10.1186/s12876-026-05119-3

**Published:** 2026-07-23

**Authors:** Srikanth Thiyagarajan, Mahalakshmi N, Abhishek Reji

**Affiliations:** Department of general surgery, SRM Medical College Hospital and Research Centre, Faculty of medicine and health sciences, SRM institute of science and Technology, Kattankulathur, Chengalpattu, Tamil Nadu India

**Keywords:** Gastric carcinoma, Skeletal muscle metastasis, Intramuscular metastasis, Adenocarcinoma, Rare metastasis, Systematic review

## Abstract

**Background:**

Skeletal muscle metastasis (SMM) from gastric carcinoma is exceptionally rare despite the extensive vascularity and mass of skeletal muscles. The true prevalence remains unknown, and existing knowledge is limited to isolated case reports.

**Objective:**

To systematically review all reported cases of SMM arising from gastric carcinoma, summarize clinical features, diagnostic approaches, imaging characteristics, treatments, and outcomes, and highlight gaps in existing literature.

**Methods:**

A comprehensive literature search was conducted across PubMed, Embase, Scopus, and Google Scholar from inception to January 2025, following PRISMA guidelines. All case reports or case series describing biopsy-confirmed skeletal muscle metastasis from gastric carcinoma were included.

**Results:**

A total of 31 fully documented cases were identified from 30 published studies spanning 1960–2025. Patients ranged in age from 30 to 80 years, with a male predominance. Skeletal muscle metastases most commonly involved the thigh, gluteal, paraspinal, and abdominal wall muscles. Patients typically presented with painful, enlarging intramuscular masses that were frequently misdiagnosed as abscesses or soft-tissue sarcomas. Magnetic resonance imaging (MRI) and positron emission tomography–computed tomography (PET-CT) were the most sensitive imaging modalities, while histopathological examination remained the gold standard for diagnosis. Most lesions were associated with poorly differentiated adenocarcinoma or signet-ring cell carcinoma. Survival data were available for 18 of the 31 patients; among these, the mean survival following the diagnosis of skeletal muscle metastasis was 7.7 months, with a median survival of 4.0 months (range, 0.6–30 months), indicating a poor overall prognosis.

**Conclusion:**

SMM from gastric carcinoma is extremely rare but Despite its rarity, skeletal muscle metastasis should be considered in patients with gastric carcinoma presenting with new painful intramuscular masses, particularly during postoperative surveillance. Early recognition using MRI or PET-CT followed by histopathological confirmation may facilitate timely multidisciplinary management, although the prognosis remains poor.

**Supplementary Information:**

The online version contains supplementary material available at https://doi.org/10.1186/s12876-026-05119-3.

## Introduction

Gastric carcinoma remains a major global health burden, ranking fifth in incidence and fourth in cancer-related mortality. Metastatic spread commonly involves the liver, lymph nodes, peritoneum, lung, and bone. However, skeletal muscle metastasis (SMM) is exceedingly rare, reported in less than 1% of all metastatic cancers and even rarer in gastric cancer.

Skeletal muscles constitute nearly 40% of total body mass and receive abundant blood supply, yet metastasis to these tissues is remarkably uncommon. Proposed protective mechanisms include contractile activity, high tissue pressure, lactic acid accumulation, and an active immune environment.

Since the first recorded case in the 1960s, only isolated case reports have been published. A systematic understanding of SMM from gastric carcinoma is lacking.

This review consolidates all available evidence over six decades to define clinical patterns, diagnostic considerations, treatment outcomes, and areas for future research.

## Methods

This review followed the PRISMA (Preferred Reporting Items for Systematic Reviews and Meta-Analyses) guidelines.

### Data sources

This systematic review was conducted in accordance with the PRISMA 2020 statement. A comprehensive literature search was performed across PubMed, Embase, Scopus, and Google Scholar from database inception to January 2025.In studies reporting more than one patient, each case was extracted and analyzed individually.

### Search terms

The search strategy included combinations of keywords and Boolean operators such as: (“gastric cancer” OR “gastric carcinoma”) AND (“skeletal muscle metastasis” OR “intramuscular metastasis” OR “muscle metastasis”).

Two independent reviewers screened titles, abstracts, and full texts. Discrepancies were resolved by consensus.

### Inclusion criteria

Studies were considered eligible for inclusion if they reported histologically confirmed gastric carcinoma with radiologically and/or biopsy-confirmed skeletal muscle metastasis. Case reports, case series, and observational studies describing such presentations were included in the review.

### Exclusion criteria

Studies were excluded if they described metastases involving non-skeletal muscle structures such as the myocardium or diaphragm, reported cases with an unknown primary tumor, or involved metastases arising from esophageal or gastroesophageal junction malignancies unless the primary tumor was explicitly confirmed to be gastric in origin.

### Data extraction

From all eligible reports, relevant clinical and demographic data were extracted when available. The extracted variables included patient age and sex, the specific skeletal muscle involved, presence of metastases at other sites, treatment modalities administered for the primary gastric tumor, treatment directed toward the skeletal muscle lesion, and survival duration following the diagnosis of skeletal muscle metastasis.For descriptive analyses, summary statistics were calculated using only cases with available data for the relevant variable. Cases with missing information were excluded from the denominator for that specific analysis. A total of 30 studies describing 31 patients were included in the final analysis. The demographic characteristics, metastatic sites, treatment modalities, and survival outcomes of the included patients are summarized in Table 1.

**Table 1:** Clinical characteristics of reported cases of skeletal muscle metastasis from gastric carcinoma (*n* = 31 patients from 30 studies)

S.NO	Author	Year	Age	Sex	Muscle Site of Metastasis	Timing	Interval to SMM	Other site of metastasis	Treatment for gastric cancer	Treatment for muscle metastasis	Survival after the muscle metastasis diagnosis	Histopathology	Reference
1	Rosenbaum et al.	1984	54	Male	Upper arm muscle femoral muscle	Synchronous	0	No other metastasis	Radiotherapy, chemotherapy	Radiotherapy, chemotherapy	2 months	Gastric adenocarcinoma	[1]
2	Arnold et al.	1989	59	Female	Medial Rectus Muscle	Synchronous	0	No other metastasis	Not available	Not available	Not available	Gastric adenocarcinoma	[2]
3	Porlie et al.	1990	65	Male	Sartorius muscle rectus femoris muscle	Synchronous	0	No other metastasis	chemotherapy	chemotherapy		Poorly differentiated adenocarcinoma	[3]
4	Sudo et al.	1993	61	Male	Trapezius muscle	Metachronous	12	No other metastasis	Radiotherapy, chemotherapy	Radiotherapy, chemotherapy	5 months	Gastric adenocarcinoma	[4]
5	Van Gelderen	1993	47	Female	Extraocular muscle	Synchronous	0	No other metastasis	Managed conservatively	Managed conservatively	Not available	Gastric adenocarcinoma	[5]
6	Toillon et al.	1994	68	Male	Calf Muscles	Metachronous	24	Esophageal hiatus & celiac lymph nodes	Chemotherapy	Chemotherapy		Gastric adenocarcinoma	[6]
7	Amano et al.	1996	57	Male	Calf Muscles	Metachronous	18	No other metastasis	Chemotherapy	Excision + chemotherapy		Poorly differentiated adenocarcinoma	[7]
8	Narvaez et al.	1998	49	Male	Psoas muscle	Metachronous	36	No other metastasis	Not available	Not available	Not available	Gastric adenocarcinoma	[8]
9	Pestalozzi et al.	1998	72	Female	Gastrocnemius muscle	Synchronous	0	Mediastinal nodes		Excision + chemo radiotherapy		Poorly differentiated adenocarcinoma	[9]
10	Oba et al.	2001	70	Male	Left lumbar muscle, left iliopsoas muscle	Metachronous	48	Brain, liver, lungs, adrenal glands	None	none	71 days	Poorly differentiated adenocarcinoma	[10]
11	Kondo et al.	2002	64	Female	Left gluteus maximus, left adductor magnus muscle	Metachronous	12	Abdominal wall	Total gastrectomy with splenectomy	Tumor excision of left gluteal mass, chemotherapy	13 months	Signet- ring cell carcinoma	[11]
12	Touheti et al.	2004	48	Male	Gluteal muscle	Metachronous	Not available	Not available	Excision	wide excision	6 months	Not available	[12]
13	Touheti et al.	2004	89	Male	Shoulder muscle	Metachronous	Not available	Not available	Excision	Radiation therapy	10 months	Not available	[12]
14	Beşe NS et al.	2006	67	Male	Posterior right paralumbar muscle & posterior left paradorosal muscle	Synchronous	0	Perigastric and lumboaortic lymph nodes	Gastrectomy, chemoradiotherapy	Palliative chemoradiotherapy	Not available	Signet- ring cell carcinoma	[13]
15	Souayah et al.	2008	49	Male	Right lateral rectus muscle	Synchronous	0	Not Available	Radiotherapy	Radiotherapy	10 weeks	Signet- ring cell carcinoma	[14]
16	Tourgeron et al.	2009	71	Male	Deltoid muscle	Metachronous	36	None	Partial gastrectomy, chemoradiotherapy	Chemoradiotherapy	13 months	Poorly differentiated adenocarcinoma	[15]
17	Sakuma et al.	2011	64	Female	Gluteal muscle	Metachronous	84	Retroperitoneal and peritoneal dissemination	Total gastrectomy	Chemotherapy	18 months	Signet- ring cell carcinoma	[16]
18	Gogou et al.	2012	Not available	Male	Femoral muscle	Synchronous	0	No liver or lung metastases	Gastrectomy	Wide excision, radiotherapy	30 months	Poorly differentiated adenocarcinoma	[17]
19	Pergolini et al.	2014	47	Male	Adductor muscle	Synchronous	0	Widespread metastatic disease	Chemotherapy	Chemotherapy	74 days	Poorly differentiated adenocarcinoma	[18]
20	Lourenco et al.	2014	68	Male	Right thigh	Synchronous	0	None	Chemotherapy	Chemotherapy	Not available	Poorly differentiated adenocarcinoma	[19]
21	Koga et al.	2015	71	Male	Latissimus dorsi, transverse abdominal, iliopsoas, femoral muscle	Synchronous	0	None	Preoperative chemotherapy, total gastrectomy	Chemotherapy	18 days	Poorly differentiated adenocarcinoma	[20]
22	Ebisui	2016	49	Female	femoral muscle	Metachronous	79	Not available	Not available	Not available	Not available	well- differentiated adenocarcinoma	[21]
23	Temido et al.	2017	42	Male	Extraocular muscle	Synchronous	0	Bone metastasis, mediastinal and abdominal ganglia	None	None	Shortly after diagnosis	Signet- ring cell carcinoma	[22]
24	Kamitani et al.	2018	47	Male	Left latissimus dorsi, paraspinal muscle, quadriceps	Metachronous	72	None	Distal gastrectomy, chemotherapy	Chemotherapy	7 months	Poorly differentiated adenocarcinoma	[23]
25	Aguirre et al.	2019	57	Female	Obturator internus, vastus lateralis, quadratus lumborum, psoas, gluteus maximus, piriformis muscle	Synchronous	0	Peritoneal carcinomatosis	Total gastrectomy, chemoradiation	Palliative radiotherapy	3 months after discharge	Poorly differentiated signet - ring cell carcinoma adenocarcinoma	[24]
26	Goto et al.	2019	54	female	Left medial rectus muscle	Synchronous	0	Ovary and mesentery, thoracic bone marrow	Chemotherapy	Radiotherapy	3 months	Signet - ring cell carcinoma	[25]
27	Daneti et al.	2020	42	Male	Psoas muscle, gluteal muscle	Synchronous	0	Not available	Antropyloric stenting	Supportive care	6 weeks	Poorly differentiated adenocarcinoma	[26]
28	Garcia et al.	2021	44	Female	Left deltoid muscle	Synchronous	0	None	Gastrectomy, chemotherapy	Excision	Not available	Gastric adenocarcinoma	[27]
29	Sellami et al.	2022	53	Male	Right superior oblique muscle	Synchronous	0	Lungs	Chemotherapy	Chemotherapy	Not available	Signet - ring cell carcinoma	[28]
30	Roohe et al.	2021	67	Male	Left rectus lateralis muscle	Synchronous	0	Leptomeningeal metastasis	None	None	2 months	Signet - ring cell carcinoma	[29]
31	Pichioni et al.	2024	45	Female	Multiple skeletal muscles of the back and abdominal wall	Synchronous	0	Peritoneal dissemination	Chemotherapy	Chemotherapy	17 months - alive	Poorly differentiated signet - ring cell carcinoma adenocarcinoma	[30]

### PRISMA flow summary

The systematic selection process is summarized in a flow diagram detailing the progression from initial database identification to final inclusion. A total of 487 records were identified through electronic database searches of PubMed, Embase, Scopus, and Google Scholar. After removal of 165 duplicate records, 322 unique articles remained and were screened based on titles and abstracts. Following this initial screening, 33 full-text articles were assessed for eligibility according to the predefined inclusion and exclusion criteria. Of these, 30 reports met the eligibility requirements and were included in the qualitative synthesis. These reports collectively described 31 individual patients, which constituted the final study population for this systematic review.One included study (Touheti et al., 2004) reported two individual patients.

**Fig. 1:**
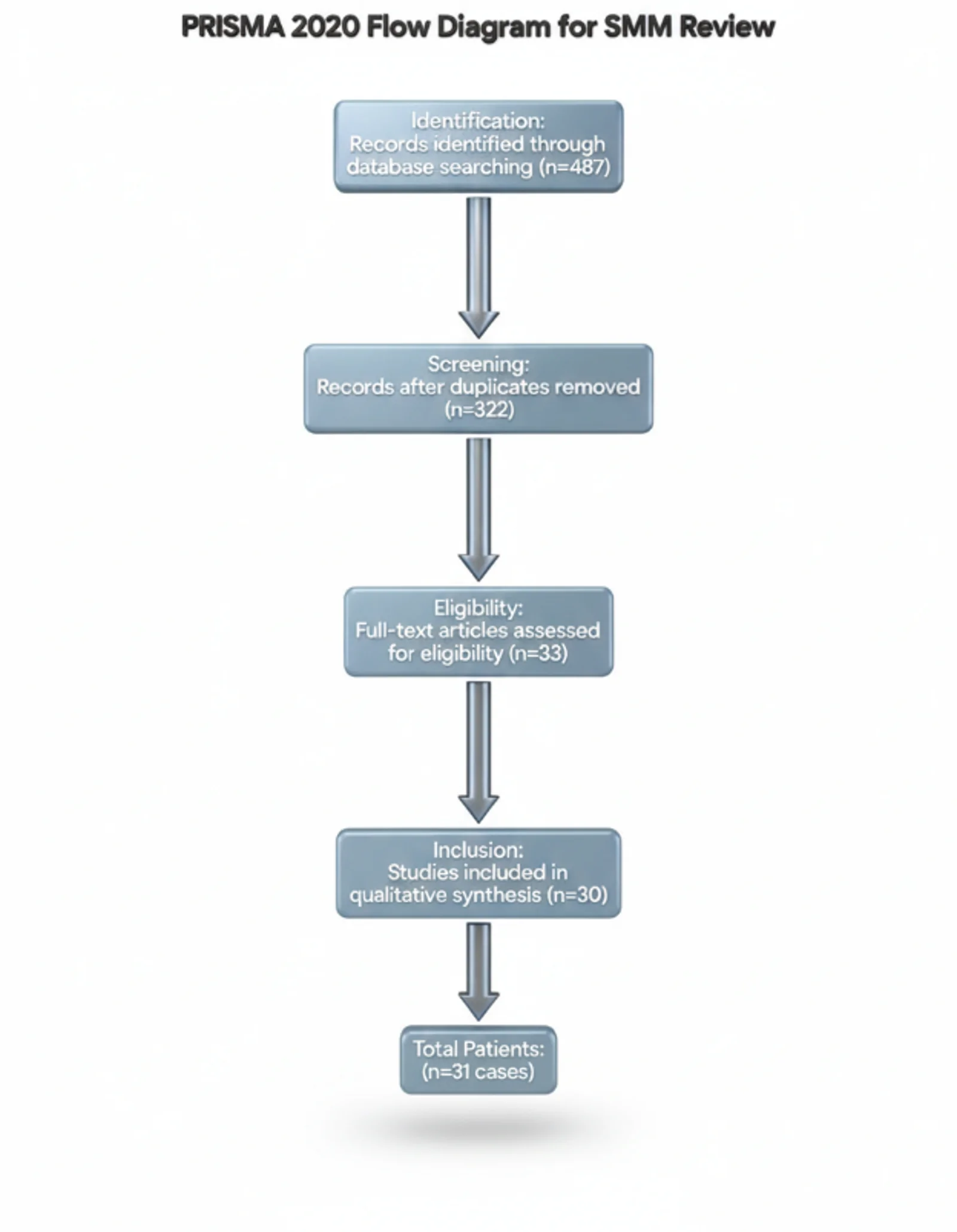
PRISMA flow diagram illustrating study selection process for inclusion in the systematic review of skeletal muscle metastasis from gastric carcinoma

Study selection was performed according to PRISMA guidelines, and the process is illustrated in Fig. 1.

### Outcome measures

The primary outcomes of interest included the clinical presentation patterns, diagnostic characteristics, treatment strategies employed, and survival outcomes following the detection of skeletal muscle metastasis.

### Quality assessment

The methodological quality of the included studies was evaluated using the Joanna Briggs Institute Checklist, and the results of the quality assessment are presented in Table 2. Each study was evaluated across eight domains, including patient demographics, clinical history, diagnostic clarity, intervention details, outcomes, and follow-up.

The overall quality of included studies was variable. Older case reports frequently lacked detailed clinical information, diagnostic clarity, and follow-up data, resulting in lower scores (0–3/8). In contrast, more recent studies demonstrated moderate to high quality (scores 5–7/8), with comprehensive reporting of clinical presentation, imaging findings, treatment, and outcomes.The distribution of studies according to methodological quality categories is summarized in Table 3.

**Table 2:** Methodological quality assessment of included case reports using the Joanna Briggs Institute Checklist (*n* = 31)

S.No	Study	Score	Quality
1	Rosenbaum 1984 [1]	6	Moderate
2	Arnold 1989 [2]	4	Moderate
3	Porlie 1990 [3]	6	Moderate
4	Sudo 1993 [4]	7	High
5	Van Gelderen 1993 [5]	6	Moderate
6	Toillon 1994 [6]	6	Moderate
7	Amano 1996 [7]	7	High
8	Narvaez 1998 [8]	4	Moderate
9	Pestalozzi 1998 [9]	7	High
10	Oba 2001 [10]	8	High
11	Kondo 2002 [11]	8	High
12	Touheti 2004 (1) [12]	7	High
13	Touheti 2004 (2) [12]	7	High
14	Bese 2006 [13]	6	Moderate
15	Souayah 2008 [14]	7	High
16	Tourgeron 2009 [15]	8	High
17	Sakuma 2011 [16]	8	High
18	Gogou 2012 [17]	7	High
19	Pergolini 2014 [18]	8	High
20	Lourenco 2014 [19]	6	Moderate
21	Koga 2015 [20]	8	High
22	Ebisui 2016 [21]	4	Moderate
23	Temido 2017 [22]	8	High
24	Kamitani 2018 [23]	8	High
25	Aguirre 2019 [24]	8	High
26	Goto 2019 [25]	8	High
27	Daneti 2020 [26]	8	High
28	Garcia 2021 [27]	8	High
29	Sellami 2022 [28]	8	High
30	Roohe 2022 [29]	8	High
31	Pichioni 2024 [30]	8	High

**Table 3:** Distribution of methodological quality of included studies

Quality Level	Score	No. of Studies
Low	0–3	0
Moderate	4–6	9
High	7–8	22

### Methods justification

Given the rarity of skeletal muscle metastasis from gastric carcinoma and the absence of large-scale observational studies or randomized controlled trials, a systematic review of published case reports and case series was considered the most appropriate methodological approach. This design enables comprehensive aggregation of dispersed evidence to identify clinical patterns, diagnostic features, treatment strategies, and outcomes associated with this uncommon metastatic presentation.

## Results

### Overview

The study selection process is summarized in Fig. 1. Thirty studies describing 31 patients were included in the final qualitative synthesis.

### Patient characteristics and histology

The 31 reported patients with skeletal muscle metastasis (SMM) from gastric carcinoma had a mean age of approximately 58 years (range, 30–80 years), with a clear male predominance. Histopathological information was available for the majority of reported cases. Poorly differentiated adenocarcinoma and signet-ring cell carcinoma were the predominant histological subtypes, indicating that skeletal muscle metastasis is more frequently associated with biologically aggressive gastric cancers. Histological subtype was unavailable in several earlier case reports, reflecting variations in reporting practices over the study period.

### Timing of metastasis

Based on the interval between the diagnosis of the primary gastric carcinoma and the detection of skeletal muscle metastasis, two distinct patterns were observed. Metachronous metastases were more frequently reported, among patients with metachronous disease, the interval between the diagnosis (or curative treatment) of the primary gastric carcinoma and the development of skeletal muscle metastasis ranged from several months to 84 months, demonstrating that skeletal muscle recurrence may occur long after apparently successful treatment, whereas synchronous metastases were identified at the time of the initial diagnosis. This variability highlights the need for continued clinical vigilance for skeletal muscle metastasis throughout the disease course, particularly in patients presenting with new painful intramuscular masses.

### Common muscle sites

Skeletal muscle metastasis occurred at a wide range of anatomical sites. The most frequently involved locations were the lower limb muscles, including the calf, soleus, gastrocnemius, quadriceps, vastus lateralis, psoas, iliacus, sartorius, and gluteal muscles. Metastatic involvement was also reported in upper limb and trunk muscles such as the deltoid, trapezius, latissimus dorsi, and abdominal wall musculature. In addition, several cases demonstrated involvement of extraocular muscles, including the medial and lateral rectus and superior oblique muscles, as well as paraspinal and lumbar muscles. This broad anatomical distribution underscores the importance of maintaining a high index of clinical suspicion when evaluating patients with unexplained skeletal muscle swelling or pain.

### Clinical presentation

Patients most commonly presented with a painful or firm enlarging skeletal muscle mass. In some cases, the presentation mimicked an acute inflammatory process, resembling conditions such as abscess or myositis. When extraocular muscles were involved, neurological manifestations such as diplopia were observed. In rare instances, skeletal muscle metastasis represented the initial manifestation of gastric carcinoma, as reported in several cases. Owing to these nonspecific clinical features, misdiagnosis was frequent, with lesions often initially interpreted as abscesses, soft-tissue sarcomas, or hematomas.

### Associated metastasis

Many patients exhibited synchronous systemic dissemination at the time of diagnosis of skeletal muscle metastasis, most commonly involving the peritoneum, lungs, liver, brain, and regional or distant lymph nodes. However, several reports described isolated or metachronous skeletal muscle metastasis without evidence of concurrent visceral involvement, including cases affecting the calf or thigh musculature. These presentations suggest that skeletal muscle metastasis may occasionally occur as a solitary or delayed metastatic manifestation.

### Diagnostic workup

Magnetic resonance imaging was the most sensitive diagnostic modality, typically demonstrating heterogeneous intramuscular masses with peripheral rim enhancement and surrounding edema. Positron emission tomography–computed tomography was highly effective in identifying multifocal metastatic disease owing to intense fluorodeoxyglucose uptake within metastatic lesions. Definitive diagnosis was established by tissue biopsy, which commonly revealed immunohistochemical positivity for cytokeratin 7 and CDX2 in intestinal-type gastric adenocarcinoma. In addition, atypical radiologic features such as heterotopic ossification within skeletal muscle metastases were described in some reports.

### Treatment approaches

Given the frequently disseminated nature of disease at presentation, systemic chemotherapy—most commonly platinum-based regimens combined with fluoropyrimidines—was the primary treatment modality in the majority of reported cases. Palliative radiotherapy was employed for local symptom control, particularly for pain relief. Surgical excision of skeletal muscle lesions was undertaken in selected patients, either for diagnostic confirmation or for palliative purposes. In more recent reports, immunotherapeutic approaches, including HER2-targeted therapy and programmed death ligand-1 inhibitors, were utilized in selected cases based on tumor molecular characteristics.

### Prognosis

Survival outcomes following the diagnosis of skeletal muscle metastasis suggest that this manifestation generally represents a terminal stage of disease, although occasional outliers have been reported. Among patients with available follow-up (*n* = 18), the mean survival after diagnosis of skeletal muscle metastasis was 7.7 months, while the median survival was 4.0 months (range 0.6–30 months). The longest reported survival was 30 months, as described by Gogou et al. These findings highlight the importance of early recognition—particularly through sensitive imaging modalities such as magnetic resonance imaging or positron emission tomography–computed tomography—to facilitate timely transition to appropriate palliative care strategies.

## Discussion

### Interpretation

Skeletal muscle metastasis from gastric adenocarcinoma is a rare but important clinical entity. The large number of muscles reported—extraocular to gluteal—highlights that metastasis can occur in any skeletal muscle group. The heterogeneity in presentation often leads to misdiagnosis as abscess, soft-tissue sarcoma, thrombophlebitis, or benign inflammatory conditions.

The rarity of skeletal muscle metastasis, despite high vascularity, remains a paradox in oncology. This review consolidates over six decades of evidence and highlights that such metastases may not be as incidental as previously thought but rather underreported due to diagnostic challenges.

The predominance of metastases involving the thigh and gluteal muscles observed in this review may reflect their relatively large muscle mass and abundant vascular perfusion, increasing the likelihood of tumour cell entrapment during haematogenous dissemination. Nevertheless, no specific skeletal muscle group appears to be selectively predisposed, and metastases have been reported in diverse anatomical locations, emphasizing the need for clinicians to maintain a high index of suspicion when evaluating new painful intramuscular lesions in patients with current or previous gastric carcinoma.

### Pathophysiology of resistance

Several physiological mechanisms have been proposed to explain the relative resistance of skeletal muscle to metastatic involvement. The unique metabolic environment of skeletal muscle, characterized by high concentrations of lactic acid and fluctuating oxygen levels, may result in a locally acidic milieu that inhibits tumor cell proliferation or induces apoptosis. In addition, constant muscle contractility and elevated intramuscular pressure generate mechanical forces that create an unfavorable environment for stable implantation and growth of metastatic cells. Furthermore, skeletal muscle exhibits active immune surveillance, with local production of anti-tumor cytokines and immune mediators that may further suppress metastatic colonization.

The predominance of metachronous skeletal muscle metastasis highlights the importance of continued oncological surveillance even after apparently curative treatment of the primary gastric tumour. In several cases, skeletal muscle involvement developed months or years after gastrectomy, suggesting that dormant micrometastatic disease may persist despite initial disease control. Consequently, new painful or enlarging intramuscular masses in patients with a history of gastric carcinoma should prompt consideration of metastatic disease, regardless of the interval since primary treatment.

### Diagnostic challenges

Although MRI provides excellent soft-tissue characterization and PET-CT facilitates the detection of metabolically active lesions throughout the body, imaging findings alone are frequently insufficient to distinguish skeletal muscle metastases from abscesses, primary soft-tissue sarcomas, or inflammatory myopathies. Therefore, histopathological confirmation through image-guided core needle biopsy or excisional biopsy remains essential for establishing the diagnosis and guiding subsequent management.

### Therapeutic implications

At present, there is no standardized treatment protocol for skeletal muscle metastasis from gastric adenocarcinoma. Systemic chemotherapy, most commonly platinum-based regimens combined with fluoropyrimidines, remains the cornerstone of management due to the frequent presence of disseminated disease. Palliative modalities, including radiotherapy for pain control and surgical excision for isolated or symptomatic lesions, play an important supportive role. In selected contemporary cases, targeted and immunotherapeutic strategies—such as HER2-directed therapy and programmed death ligand-1 inhibitors—have been employed based on tumor molecular profiling.

### Prognosis

The poor survival observed in this review likely reflects the presence of advanced systemic disease rather than the skeletal muscle metastasis itself. Skeletal muscle involvement is commonly accompanied by widespread metastatic dissemination, limiting the effectiveness of local therapies. The longest interval to skeletal muscle metastasis identified in this review was seven years, illustrating the unpredictable biological behaviour of gastric carcinoma and reinforcing the need for continued vigilance beyond conventional surveillance periods.Consequently, treatment is generally individualized, with systemic chemotherapy forming the cornerstone of management, while surgery or radiotherapy may be considered for selected patients with isolated symptomatic lesions or for palliative purposes.

### Methodology defence

The present study adopts a systematic review approach focusing on case reports and small case series, which represents the highest level of available evidence for rare clinical entities such as skeletal muscle metastasis from gastric carcinoma. Although such evidence is inherently descriptive, it provides valuable insights into clinical presentation patterns, diagnostic challenges, and therapeutic strategies that cannot be captured through conventional large-scale studies due to the extremely low incidence of this condition.

Despite inherent limitations, systematic aggregation of rare case-based evidence remains essential for improving clinical awareness and guiding decision-making in uncommon oncological presentations.

## Limitations

This review has several limitations. First, the available evidence consists exclusively of case reports and small case series, making the findings susceptible to publication and reporting bias. Second, the rarity of skeletal muscle metastasis from gastric carcinoma resulted in a limited sample size, precluding quantitative meta-analysis and restricting the statistical power of subgroup analyses. Third, several reports lacked complete information regarding histopathological subtype, immunohistochemical findings, interval to metastasis, treatment details, and follow-up duration, which limited the precision of pooled estimates. Additionally, variations in diagnostic techniques, therapeutic strategies, and reporting standards over the six-decade study period introduced considerable clinical heterogeneity. Finally, survival analyses were based only on patients with available follow-up data, and several cases reported ongoing treatment or incomplete follow-up, potentially influencing the calculated survival estimates.

Future studies should focus on the establishment of multicentre registries and collaborative databases to facilitate the systematic collection of clinicopathological characteristics, molecular profiles, treatment strategies, and long-term outcomes of patients with skeletal muscle metastasis from gastric carcinoma. Standardized reporting of rare metastatic presentations will improve understanding of disease biology, enable more robust prognostic analyses, and help develop evidence-based recommendations for diagnosis and management.

## Key clinical insights

- Poorly differentiated adenocarcinoma and signet-ring cell carcinoma were the predominant histological subtypes associated with skeletal muscle metastasis.
- Metachronous presentation was more common than synchronous presentation, with recurrence occurring up to seven years after treatment of the primary tumour.
- MRI and PET-CT were the most useful imaging modalities, but histopathological confirmation remained essential.
- Mean survival was 7.7 months (median 4.0 months) among patients with available follow-up data, reflecting the aggressive nature of this disease.

## Conclusion

Skeletal muscle metastasis from gastric carcinoma represents a rare but diagnostically challenging manifestation of advanced disease. Early recognition using high-resolution imaging and timely biopsy is essential to avoid misdiagnosis. Future multicenter registries and molecular studies are required to elucidate mechanisms of muscle tropism and improve therapeutic strategies.

While most cases represent advanced, disseminated disease with a poor prognosis, early recognition is vital for optimizing palliative strategies and improving the patient’s quality of life. Future research utilizing international registries and molecular profiling is needed to better understand the mechanisms of muscle tropism and evaluate the efficacy of modern immunotherapies.

## Supplementary Information

Supplementary Material 1.

## Data Availability

All data supporting the findings of this study are available within the paper and its Supplementary Information.

## Abbreviations

CRT: Chemoradiotherapy
CT: Computed Tomography
JBI: Joanna Briggs Institute Checklist
LN: Lymph Node
MRI: Magnetic Resonance Imaging
NA: Not Available
PET: Positron Emission Tomography
PRISMA: Preferred Reporting Items for Systematic Reviews and Meta–Analyses
RT: Radiotherapy
SMM: Skeletal muscle metastasis
