# Supplementary material for: Skeletal muscle metastasis from gastric carcinoma: a systematic review of published case reports and case series over six decades
Source: BMC Gastroenterol. 2026 Jul 23;26:675. doi: 10.1186/s12876-026-05119-3 (PMC13682083; doi:10.1186/s12876-026-05119-3)
Supplement: Supplementary file 1 [file 12876_2026_5119_MOESM1_ESM.docx]

| **S.No** | **Study** | **D1** | **D2** | **D3** | **D4** | **D5** | **D6** | **D7** | **D8** | **Score** |
| --- | --- | --- | --- | --- | --- | --- | --- | --- | --- | --- |
| 1 | Rosenbaum 1984 | ✔ | ✔ | ✔ | ✔ | ✔ | ❌ | ✔ | ✔ | 6 |
| 2 | Arnold 1989 | ✔ | ✔ | ✔ | ❌ | ❌ | ❌ | ✔ | ❌ | 4 |
| 3 | Porlie 1990 | ✔ | ✔ | ✔ | ✔ | ✔ | ❌ | ✔ | ❌ | 6 |
| 4 | Sudo 1993 | ✔ | ✔ | ✔ | ✔ | ✔ | ❌ | ✔ | ✔ | 7 |
| 5 | Van Gelderen 1993 | ✔ | ✔ | ✔ | ✔ | ✔ | ❌ | ✔ | ❌ | 6 |
| 6 | Toillon 1994 | ✔ | ✔ | ✔ | ✔ | ✔ | ❌ | ✔ | ❌ | 6 |
| 7 | Amano 1996 | ✔ | ✔ | ✔ | ✔ | ✔ | ❌ | ✔ | ✔ | 7 |
| 8 | Narvaez 1998 | ✔ | ✔ | ✔ | ❌ | ❌ | ❌ | ✔ | ❌ | 4 |
| 9 | Pestalozzi 1998 | ✔ | ✔ | ✔ | ✔ | ✔ | ❌ | ✔ | ✔ | 7 |
| 10 | Oba 2001 | ✔ | ✔ | ✔ | ✔ | ✔ | ✔ | ✔ | ✔ | 8 |
| 11 | Kondo 2002 | ✔ | ✔ | ✔ | ✔ | ✔ | ✔ | ✔ | ✔ | 8 |
| 12 | Touheti 2004 (1) | ✔ | ✔ | ✔ | ✔ | ✔ | ❌ | ✔ | ✔ | 7 |
| 13 | Touheti 2004 (2) | ✔ | ✔ | ✔ | ✔ | ✔ | ❌ | ✔ | ✔ | 7 |
| 14 | Bese 2006 | ✔ | ✔ | ✔ | ✔ | ✔ | ❌ | ✔ | ❌ | 6 |
| 15 | Souayah 2008 | ✔ | ✔ | ✔ | ✔ | ✔ | ❌ | ✔ | ✔ | 7 |
| 16 | Tourgeron 2009 | ✔ | ✔ | ✔ | ✔ | ✔ | ✔ | ✔ | ✔ | 8 |
| 17 | Sakuma 2011 | ✔ | ✔ | ✔ | ✔ | ✔ | ✔ | ✔ | ✔ | 8 |
| 18 | Gogou 2012 | ✔ | ✔ | ✔ | ✔ | ✔ | ❌ | ✔ | ✔ | 7 |
| 19 | Pergolini 2014 | ✔ | ✔ | ✔ | ✔ | ✔ | ✔ | ✔ | ✔ | 8 |
| 20 | Lourenco 2014 | ✔ | ✔ | ✔ | ✔ | ✔ | ❌ | ✔ | ❌ | 6 |
| 21 | Koga 2015 | ✔ | ✔ | ✔ | ✔ | ✔ | ✔ | ✔ | ✔ | 8 |
| 22 | Ebisui 2016 | ✔ | ✔ | ✔ | ❌ | ❌ | ❌ | ✔ | ❌ | 4 |
| 23 | Temido 2017 | ✔ | ✔ | ✔ | ✔ | ✔ | ✔ | ✔ | ✔ | 8 |
| 24 | Kamitani 2018 | ✔ | ✔ | ✔ | ✔ | ✔ | ✔ | ✔ | ✔ | 8 |
| 25 | Aguirre 2019 | ✔ | ✔ | ✔ | ✔ | ✔ | ✔ | ✔ | ✔ | 8 |
| 26 | Goto 2019 | ✔ | ✔ | ✔ | ✔ | ✔ | ✔ | ✔ | ✔ | 8 |
| 27 | Daneti 2020 | ✔ | ✔ | ✔ | ✔ | ✔ | ✔ | ✔ | ✔ | 8 |
| 28 | Garcia 2021 | ✔ | ✔ | ✔ | ✔ | ✔ | ✔ | ✔ | ✔ | 8 |
| 29 | Sellami 2022 | ✔ | ✔ | ✔ | ✔ | ✔ | ✔ | ✔ | ✔ | 8 |
| 30 | Roohe 2022 | ✔ | ✔ | ✔ | ✔ | ✔ | ✔ | ✔ | ✔ | 8 |
| 31 | Pichioni 2024 | ✔ | ✔ | ✔ | ✔ | ✔ | ✔ | ✔ | ✔ | 8 |

## **Supplementary Table 1 : Quality Assessment of Included Case Reports (n = 31)**

D1: Patient demographics

D2: Clinical history and timeline

D3: Diagnostic methods

D4: Intervention details

D5: Post-intervention outcomes

D6: Adverse events

D7: Key clinical takeaway

D8: Follow-up adequacy

# **PRISMA 2020 CHECKLIST TABLE**

## **Supplementary Table 2 . PRISMA 2020 Checklist**

| **Section** | **Item No.** | **Checklist Item** | **Reported (Yes/No)** | **Location in Manuscript** |
| --- | --- | --- | --- | --- |
| Title | 1 | Identify the report as a systematic review | Yes | Title |
| Abstract | 2 | Provide a structured summary | Yes | Abstract |
| Introduction | 3 | Describe rationale for the review | Yes | Introduction |
| Introduction | 4 | Provide explicit objectives | Yes | Introduction |
| Methods | 5 | Specify inclusion and exclusion criteria | Yes | Methods 2.3–2.4 |
| Methods | 6 | Specify information sources | Yes | Methods 2.1 |
| Methods | 7 | Present full search strategy | Yes | Methods 2.2 + Supplement |
| Methods | 8 | Describe selection process | Yes | Methods + PRISMA Figure |
| Methods | 9 | Describe data collection process | Yes | Methods 2.5 |
| Methods | 10 | List data items extracted | Yes | Methods 2.5 |
| Methods | 11 | Describe risk of bias assessment | Yes | Methods 2.8 |
| Methods | 12 | Specify effect measures | No | Not applicable |
| Methods | 13 | Describe synthesis methods | Yes | Methods |
| Methods | 14 | Describe reporting bias assessment | No | Not assessed |
| Methods | 15 | Describe certainty assessment | No | Not assessed |
| Results | 16 | Describe study selection | Yes | Results + Figure 1 |
| Results | 17 | Cite included studies and characteristics | Yes | Table 1 |
| Results | 18 | Present risk of bias results | Yes | Table 2 |
| Results | 19 | Present results of individual studies | Yes | Results |
| Results | 20 | Present results of synthesis | Yes | Results |
| Results | 21 | Present reporting bias assessment | No | Not assessed |
| Results | 22 | Present certainty of evidence | No | Not assessed |
| Discussion | 23 | Interpret results in context | Yes | Discussion |
| Discussion | 24 | Discuss limitations | Yes | Limitations |
| Discussion | 25 | Discuss implications | Yes | Conclusion |
| Other | 26 | Provide registration details | No | Not registered |
| Other | 27 | Provide protocol access | No | Not available |
| Other | 28 | Describe funding sources | Yes | Declarations |
| Other | 29 | Declare competing interests | Yes | Declarations |
| Other | 30 | Describe data availability | Yes | Declarations |
